# Emissions of methane from northern peatlands: a review of management impacts and implications for future management options

**DOI:** 10.1002/ece3.2469

**Published:** 2016-09-13

**Authors:** Mohamed Abdalla, Astley Hastings, Jaak Truu, Mikk Espenberg, Ülo Mander, Pete Smith

**Affiliations:** ^1^ Institute of Biological and Environmental Sciences University of Aberdeen Aberdeen UK; ^2^ Department of Geography Institute of Ecology and Earth Sciences University of Tartu Tartu Estonia; ^3^ Hydrosystems and Bioprocesses Research Unit National Research Institute of Science and Technology for Environment and Agriculture (Irstea) Antony Cedex France

**Keywords:** bog, drainage, fen, methane emissions, natural peatlands, restoration

## Abstract

Northern peatlands constitute a significant source of atmospheric methane (CH_4_). However, management of undisturbed peatlands, as well as the restoration of disturbed peatlands, will alter the exchange of CH_4_ with the atmosphere. The aim of this systematic review and meta‐analysis was to collate and analyze published studies to improve our understanding of the factors that control CH_4_ emissions and the impacts of management on the gas flux from northern (latitude 40° to 70°N) peatlands. The analysis includes a total of 87 studies reporting measurements of CH_4_ emissions taken at 186 sites covering different countries, peatland types, and management systems. Results show that CH_4_ emissions from natural northern peatlands are highly variable with a 95% CI of 7.6–15.7 g C m^−2^ year^−1^ for the mean and 3.3–6.3 g C m^−2 ^year^−1^ for the median. The overall annual average (mean ± *SD*) is 12 ± 21 g C m^−2 ^year^−1^ with the highest emissions from fen ecosystems. Methane emissions from natural peatlands are mainly controlled by water table (WT) depth, plant community composition, and soil pH. Although mean annual air temperature is not a good predictor of CH_4_ emissions by itself, the interaction between temperature, plant community cover, WT depth, and soil pH is important. According to short‐term forecasts of climate change, these complex interactions will be the main determinant of CH_4_ emissions from northern peatlands. Drainage significantly (*p* < .05) reduces CH_4_ emissions to the atmosphere, on average by 84%. Restoration of drained peatlands by rewetting or vegetation/rewetting increases CH_4_ emissions on average by 46% compared to the original premanagement CH_4_ fluxes. However, to fully evaluate the net effect of management practice on the greenhouse gas balance from high latitude peatlands, both net ecosystem exchange (NEE) and carbon exports need to be considered.

## 1 Introduction

The concentration of methane (CH_4_) in the atmosphere has increased from 722 ppb during the pre‐industrial period to 1,819 ppb in 2012, due to increased anthropogenic emissions (Ciais et al., 2013; Whalen, 2005). Methane is the second most important greenhouse gas (GHG) after carbon dioxide (CO_2_). Although it contributes less than 0.5% of the atmospheric carbon (C) gas concentration, it constitutes about 20% of the global radiative forcing (IPCC, 2013). This is because CH_4_ has a much stronger radiative forcing (i.e., is 34 times stronger than CO_2_) (IPCC, 2013). For an emission pulse of similar mass of C, CH_4_ creates a powerful immediate radiative forcing at the start, but due to its shorter atmospheric lifetime, this declines faster than for CO_2_ (IPCC, 2013).

Globally, CH_4_ emissions are about 500–600 Tg CH_4_ per year (Bruhwiler et al., 2014; Kirschke et al., 2013). Approximately, 40% of these emissions are from natural sources, mainly wetlands, while the rest (60%) are due to microbial emissions in rice agriculture, livestock grazing and waste, biomass burning, and fossil fuel (Denman et al., 2007). Northern peatlands (i.e., latitude 40^°^–70°N) emit about 36 Tg CH_4_‐C per year (Zhuang et al., 2006), which is equivalent to 11% of the total CH_4_ emissions (Wuebbles & Hayhoe, 2002).

In wetland soils, CH_4_ is produced in the anaerobic zones of submerged soils by methanogens, is oxidized to CO_2_ by methanotrophs in the aerobic zones, and is emitted to the atmosphere when the balance between the production and consumption is positive (Le Mer & Rodger, 2001). Peat soil accumulation derives from a positive water balance and a water table (WT) close to the soil surface, which results in anaerobic conditions preserving organic material (Belyea & Clymo, 2001; Lai, 2009), and that is also a prerequisite for methanogenesis, the terminal step of anaerobic organic matter mineralization (Hou, Wang, Chen, & Patrick, 2000; Yavitt & Williams, 2000). Both the CH_4_ directly produced in the peat soil and atmospheric CH_4_ can be oxidized as an energy source, or used for biosynthesis by methanotrophs (Conrad, 1996; Hanson & Hanson, 1996).

In a recent review, Turetsky et al. (2014) concluded that the CH_4_ flux from fens is more sensitive to the vegetation type present and less sensitive to soil temperature than fluxes from bog or swamp ecosystems. Water table depth and temperature are the major controls on CH_4_ emissions from natural bogs and swamps, but other processes like vascular transport in plants could partially override the effect of these controls in other wetland types, for example, fens. Other previous studies have identified many environmental factors that exert significant control over CH_4_ emissions from peatlands, including microtopography and plant species composition (Bubier, Moore, & Roulet, 1993; Nilsson et al., 2001), temperature (Ding & Cai, 2007; Granberg, Mikkelä, Sundh, Svensson, & Nilsson, 1997; Saarnio et al., 1998), WT depth and soil moisture (Frenzel & Karofeld, 2000; Granberg et al., 1997; Hargreaves & Fowler, 1998; Liblik, Moore, Bubier, & Robinson, 1997; Moore & Knowles, 1989; Sundh, Mikkela, Nilsson, & Svensson, 1995; Yang et al., 2006), atmospheric N deposition (Bodelier & Laanbroek, 2004; Granberg, Sundh, Svensson, & Nilsson, 2001), pH (Hutsch, 1998; Singh, Singh, & Kashyap, 1999), and availability and quality of substrate (Granberg et al., 1997; Joabsson, Christensen, & Walle′n, 1999).

Methane can also be released to the atmosphere in bubbles (ebullition) which take place when there are gas pockets in the waterlogged soil, or the dispersal of the gas is prevented by a layer of dense peat or ice (Baird, Beckwith, Waldron, & Waddington, 2004; Tokida et al., 2007). Air pressure has an important role in establishing the timing and quantity of CH_4_ ebullition (Tokida et al., 2007). In the Aapa mires (fens), CH_4_ confined under ice layers can be released in the spring thaw, representing about 11% of the annual emissions (Tokida et al., 2007). In these situations, large quantities of CH_4_ (>40 g CH_4_ m^−2^) may be released to the atmosphere over periods of minutes to hours (Glaser et al., 2004; Rosenberry, Glaser, Siegel, & Weeks, 2003), where CH_4_ bubbles are transported through the peat too fast to allow oxidation to occur. Methane can also be released to the atmosphere *via* vascular plants (Joabsson et al., 1999; King, Reeburgh, & Regli, 1998). Under anoxic conditions, vascular plants in wetlands may transport O_2_ through specialized, aerenchymatous tissues, by which CH_4_ can also be released to the atmosphere (Joabsson et al., 1999). The exchange of O_2_ and CH_4_ through vascular plants between the anoxic zone and the atmosphere may have contrasting effects on CH_4_ emissions in northern peatlands. Methane production by methanogenic archaea could be inhibited by the transport of O_2_ into otherwise anaerobic layers, or oxidized due to release of O_2_ into the rhizosphere. Due to this bypass release of CH_4_, the net emission to the atmosphere tends to increase when aerenchymatous vascular plants are present (Joabsson et al., 1999). Further, CH_4_ has low solubility in water (23–40 mg/L at 0–20°C) and could escape through sediment into the atmosphere by either diffusion or ebullition. The gas could be transported through vascular plants (Joabsson et al., 1999) or diffused slowly upward through peat soils where the methanotrophic bacteria are able to oxidize it to CO_2_. Analyzing a large UK data set on CH_4_ emissions from soils, Levy et al. (2012) found that where plant species composition data (percentage cover of aerenchymatous plant species) were available, this provided the highest explanatory power of CH_4_ fluxes to the atmosphere.

Northern peatlands represent a crucial ecosystem for regional GHG budgets because they store large amounts of C (Loisel et al., 2014). However, the ratio between decomposition and conservation of the C depends on the vegetation types present, for example, Sphagnum mosses are more resistant to decomposition compared to sedges and other vascular plants and thereby retain more C over time (Rydin & Jeglum, 2006). Peatlands can be divided into two main categories, depending on their hydrology and nutrient status. These are (1) ombrotrophic peatlands (bogs) which receive water and nutrients from atmospheric deposition and thus are acidic and poor in nutrients and (2) minerotrophic peatlands (fens) which receive water and nutrients from the surrounding mineral soils in the catchment. Nutrient status in fens varies from close to ombrotrophic nutrient‐poor conditions to mesotrophic/eutrophic conditions, mainly controlled by the ratio between the peatland and mineral soil area, and the mineral nutrient status in that catchment (Clymo, 1983). Differences between the peatland types are also reflected in vegetation composition, primary production, organic matter decomposition, and C gas emissions (Clymo, 1983; Nilsson et al., 2001). Peatlands are also classified into aquatic, forb, graminoid, lichen, moss, nonvegetated, shrub, and treed based on the general form of the vegetation cover, rather than on species (Adams et al., 1997).

Management of peatlands, through, for example, changes in land use, drainage, and cultivation of natural peatlands and application of N fertilizer disturb methanogenic archaea (Reeburg, Whalen, & Alperin, 1993) and methanotrophic bacteria (Seghers et al., 2003; Tate et al., 2007), leading to peatlands becoming a weak CH_4_ sink (Castaldi, Ermice, & Strumia, 2006; Tate et al., 2007). Large areas of northern peatland have been drained and used for agriculture, forestry, and peat extraction (Laine, Vasander, & Laiho, 1995). Peatlands are drained to lower the WT away from the surface and this has profound impacts on the functioning of the peatlands. Lowering WT by drainage results in changing biological, chemical, and physical characteristics of the soils, enhancing soil aeration (Hillman, Gerbemedhin, & Warner, 1992; Prevost, Belleau, & Plamondon, 1997) and increasing soil temperatures (Kirschbaum, 1995), thereby reducing CH_4_ emissions (Nykänen, Alm, Silvola, Tolonen, & Martikainen, 1998; Von Arnold, Nilsson, Hanell, Weslien, & Klemedtsson, 2005; Von Arnold, Weslien, Nilsson, Svensson, & Klemedtsson, 2005). On the other hand, restoration practices aim to re‐establish the conditions that encourage peat accumulation (Kimmel & Mander, 2010; Vasander et al., 2003). They include techniques to raise the WT and re‐establish vegetation cover that could enhance the waterlogged environment and enable peat accumulation to be established (Worrall et al., 2011).

Wetland restoration is one method with which northern countries could aim to meet their GHG targets under the Kyoto Protocol (Bain, Hornsey, Bongiorno, & Jeffries, 2012). In contrast to drainage, restoration raises the WT, increases water saturation, and thus may increase CH_4_ emissions (Saarino, Winiwarter, & Leitao, 2009). The WT level controls the balance between CH_4_ and CO_2_ emissions and the rate of CH_4_ emissions to the atmosphere is therefore very sensitive to WT depth (Price & Ketcheson, 2009; Sirin & Laine, 2008). The most prevalent restoration method is drain blocking, which could restore the WT to its initial state (Holden et al., 2007), or raising the WT by gully and ditch blocking (Evans, Monteith, & Cooper, 2005). Other restoration methods include planting and reseeding of bare surfaces, or re‐establishment of natural peatland vegetation, which is important, as vegetation is a major factor in peat formation (Petrone, Price, Waddington, & von Waldow, 2004; Vitt, 2006).

Predicted changes in climate, including rising temperatures, changes in the amount, intensity, and seasonal distribution of precipitation and amount of snow fall and cover (IPCC, 2013), could affect the dynamics of hydrology in northern peatlands and could increase methane production (FAO, 2008). Additionally, the exploitation of peatlands for agriculture, energy, and horticulture under intensive management also greatly influences the rate of mineralization (CO_2_ emissions) (Laine et al., 1995). Higher CH_4_ emissions could lead to a positive feedback on climate change and thereby further disturbance of peatland C stocks (Friedlingstein et al., 2006). It is suggested that climate change reduces the capacity of northern peatlands to absorb atmospheric carbon dioxide (Wu & Roulet, 2014) and this depends on how management, and the interaction with climate change, will affect CH_4_ emissions. The aim of this systematic review and meta‐analysis was to collate and analyze published studies to improve our understanding of the factors that control CH_4_ emissions and the impacts of management on the gas flux from northern peatlands. The specific hypotheses that we tested were as follows: (1) Methane emission is mainly controlled by WT, plant community, temperature, and pH; (2) management, especially drainage and restoration, significantly affects CH_4_ emissions; and (3) climate change will significantly reduce the capacity of northern peatlands to absorb the atmospheric C.

## 2 Materials and Methods

### 2.1 Data collection

**Figure 1:**
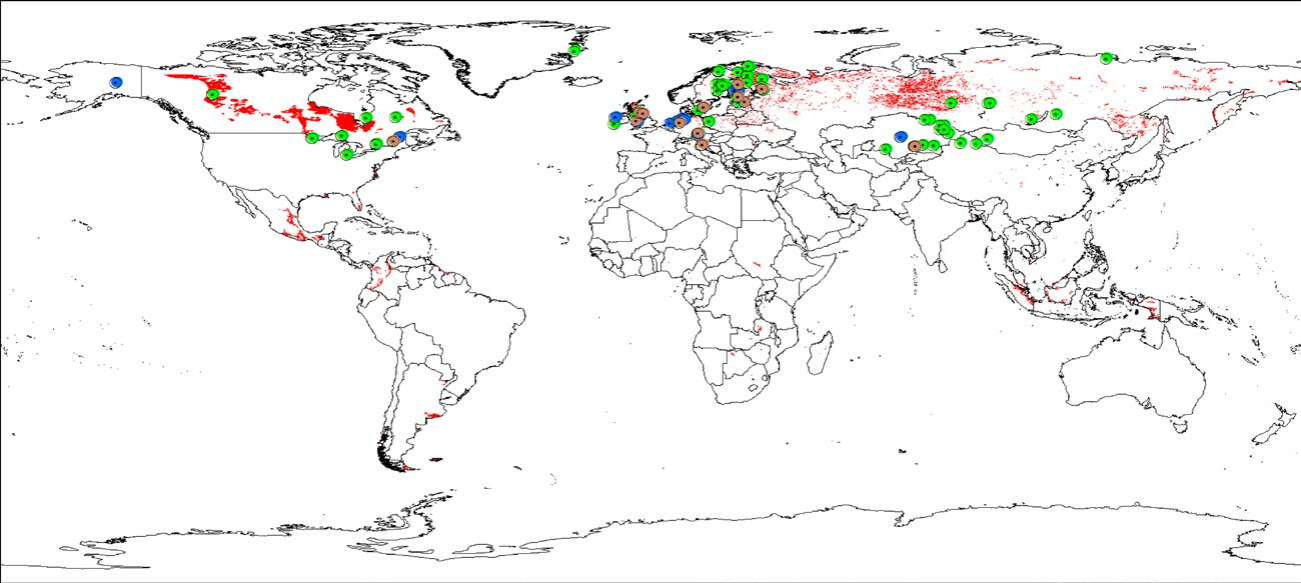
World map showing the experimental sites included in this study, across the northern peatlands. Symbols: Green = pristine, brown = drained, blue = restored; the red areas indicate histosols

To locate all papers that have reported CH_4_ emissions from northern peatlands, we performed a comprehensive search on the Web of Science database (accessed between January 2013 and July 2016) using the keywords: pristine peatlands, methane emissions, drainage, restoration, fens, bogs, mire, and northern peatlands. In an attempt to gain a comprehensive coverage, we also checked all references in the papers found in the Web of Science search. Only studies which covered at least one growing season and measured at weekly or more frequent intervals were selected. These searches resulted in 87 studies reporting measurements of CH_4_ emissions taken at 186 sites covering different countries, peatland types, and management systems (Fig. 1). To indicate the direction of the methane flux, we used the atmospheric science sign convention, that is, a negative sign represents uptake of CH_4_ gas by the ecosystem. In cases where a site has several years of flux data, the average flux of these years was used. If the flux values covered the growing season only, we estimated the annual flux values based on a previously used factor, generated from studies with full annual measurements coverage, whereby winter fluxes were estimated to constitute 15% of the annual CH_4_ fluxes (Maljanen et al., 2010; Saarino et al., 2007). All CH_4_ flux values were converted to g C m^−2^ year^−1^. The overall CH_4_ flux average ± *SD* (g C m^−2^ year^−1^) for “natural peatlands” was based on site averages reported in each publication and did not account for the variation between years at a single site. Some studies are repeated in more than one table because they include more than one site of different management systems.

For the studies included in this meta‐analysis, CH_4_ fluxes were measured using different methods which may differ in their ability to capture ebullition fluxes. These are manual chamber measurements, autochambers, and eddy covariance flux towers. Also, different methods were used to measure soil pH, for example, using pH probe/meter in deionized water or 0.01 M CaCl_2_ in 1:1 and 1:2 or 1:5 (v:v) soils: solution ratios. We assumed the pH results to be equivalent and, where a range of values were reported, we took the mean. Where air temperature was reported, we used the mean annual temperature in degree Celsius (°C) as variations between years were minor. The WT was reported relative to the surface in centimeters (cm) and we used the convention of negative values representing distance below the surface. Where a range of water levels was reported over the study, we used the mean value in the meta‐analysis. In this review, we have adopted the classification of fens/bogs, for consistency, as most of the sites included were classified into fen or bog. From the descriptions of the sites in each paper used in this study, we assigned a peatland type of either fen or bog or wooded fen and bog.

### 2.2 Data analysis

We used Minitab 16 (Minitab Inc., State College, PA, USA) and R version 3.3.0 (R Development Core Team, 2016) for data exploration, conditioning, and analysis. We split the literature studies into three groups for analysis: natural, drained, and restored. We used different analytical procedures for each group appropriate for the available published data.

#### 2.2.1 Natural peatlands

The data collected from natural peatlands covered 56 studies and 108 sites. The predictive variables available to test the response variable of annual methane flux were as follows: latitude, longitude, duration of measurement, mean annual air temperature (T), mean pH (pH), and mean WT as covariates and bog, fen, and woodland as random factors. Data exploration using matrix plots determined that latitude was colinear with mean annual air temperature as were mean pH and WT depth with their maximum and minimum values and these were excluded from the analysis. Annual precipitation, evapotranspiration, and water flow through were not available for most studies so observed WT depth was used as the explanatory variable relating to both water supply and the oxidation status. Normality in flux and residual was tested and the flux was log‐transformed. A one‐way ANOVA test was performed to test whether there was a significant different in emissions between bog, fen, and wooded peatlands. Next a linear mixed‐effects model (LMM) was applied to test annual methane flux relationships with environmental variables and type of peatland using the “lmer” method (version 1.1–12) (Bates, Mächler, Bolker, & Walker, 2014), while *p*‐values were calculated in order to confirm the significance of the relationships using the lmerTest package version 2.0–30 (Kuznetsova, Brockhoff, & Bojesen Christensen, 2013) in R version 3.3.0 (R Development Core Team, 2016). The package “piecewiseSEM” version 1.1.3 (Lefcheck and Jonathan, 2016) was used to calculate values for explained variation for obtained linear mixed‐effects models. Not all variables were available at all sites with pH being available for the least, so the LMM was performed on samples that had pH values (*n* = 36) and then repeated on samples without the variable “pH” (*n* = 76). Then, package “missMDA” version 1.10 (Josse & Husson, 2016) was applied to impute missing data values, resulting in 108 samples to which the LMM was applied for all samples and all variables. Multiple linear regression analysis was applied to estimate the variation explained by two environmental variables.

The package “akima” version 0.5–12 was used to create interpolated contour plots (Akima & Gebhardt, 2015) of pairs of the environmental parameters as x and y with annual CH_4_ emissions as the *z* variable. This was made for both the available study data and the imputed data to verify that the data trends were similar and the imputed values are valid. As WT and peatland type explained 42% of the variability, we performed linear regressions of these variables against the log‐transformed annual CH_4_ flux, with and without identified outliers. Then, we estimated the regression model of annual CH_4_ flux and mean annual water level by nonlinear least squares, using the R function “nls.”

#### 2.2.2 Drained peatlands

The data were tested, using paired *t*‐tests on all paired sites where both natural (*n* = 42) and artificially drained (*n* = 61) (i.e., lower water table for using in agriculture, forestry or mining) peatlands had CH_4_ emission measurements.

In addition, a *t*‐test was performed to see whether there was a significant difference between drained fens (*n* = 26) and drained bogs (*n* = 35). The effects of different land use systems/vegetation cover (cropland [*n* = 4], grassland [*n* = 7], and woods [*n* = 29]) on drained peatland methane emissions were also tested using one‐way ANOVA.

#### 2.2.3 Restored peatlands

The impacts of restoration system on CH_4_ emissions from peatlands were investigated. The management systems tested using paired *t*‐test were as follows: rewetting (*n* = 16), and restoring by vegetation and rewetting (*n* = 16).

## 3 Results

### 3.1 Methane emissions from northern natural peatlands

**Figure 2:**
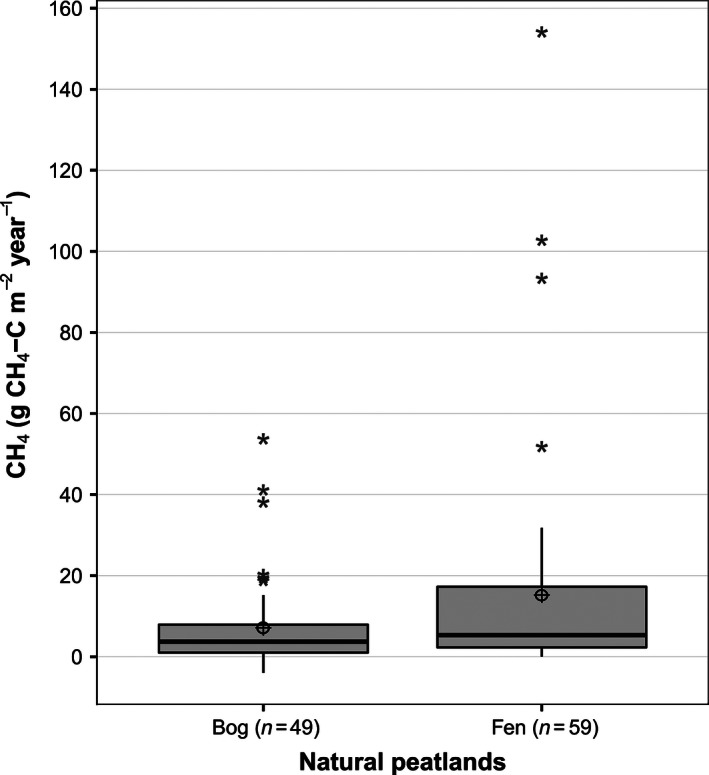
Box and whiskers plot showing median, 25 and 75% median quartiles, mean (⊕), 95% confidence interval (whiskers), and outlier (*) values of mean annual methane emissions per peatland type. *T*‐test indicates a significant difference (*t* = −1.99; *p* < .05) between the two groups

**Figure 3:**
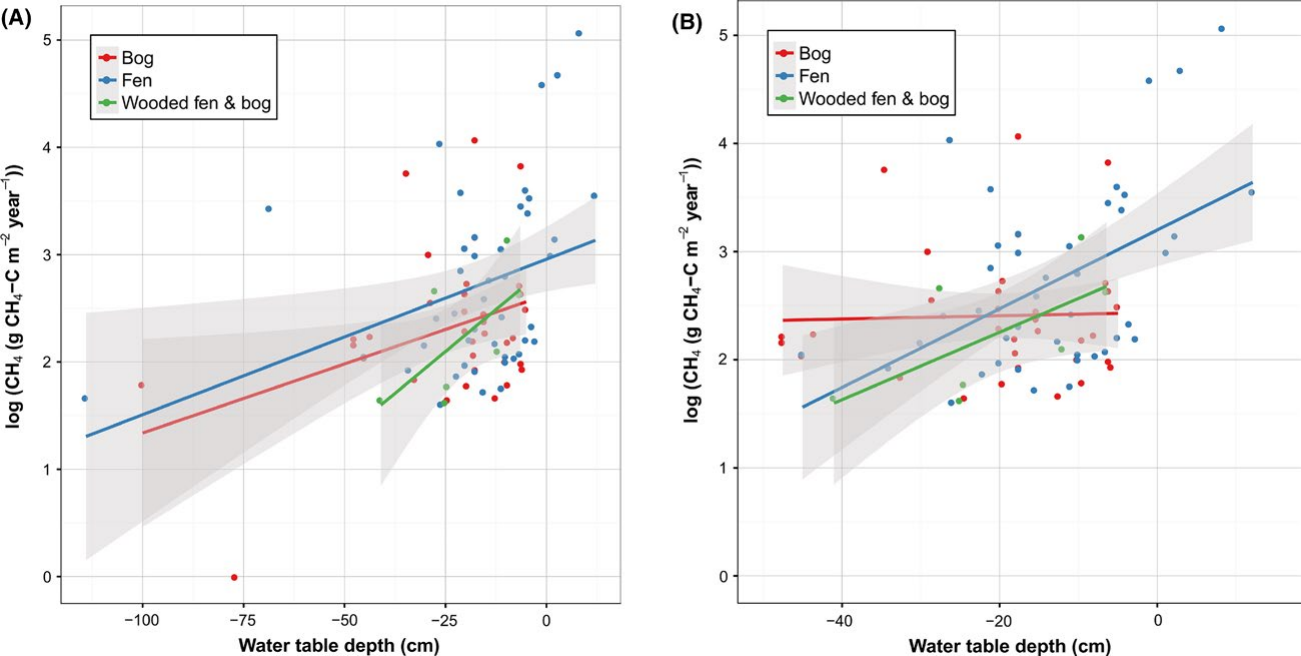
Relationships between annual CH_4_ flux and mean annual water table in different peatland types: (A) using all available data: bog (*n* = 35, *r* ^2^ = .11, *p* < .05), fen (*n* = 45, *r* ^2^ = .13, *p* < .01), and wooded fen and bog (*n* = 7, *r* ^2^ = .36, *p* = .09); (B) when 4 outliers are removed: bog (*n* = 33, *r* ^2^ = .36, *p* = .8), fen (*n* = 43, *r* ^2^ = .22, *p* < .001), and wooded fen and bog (*n* = 7, *r* ^2^ = .36, *p* = .09). The shaded area represents 95% confidence intervals of the linear regression trend lines

**Figure 4:**
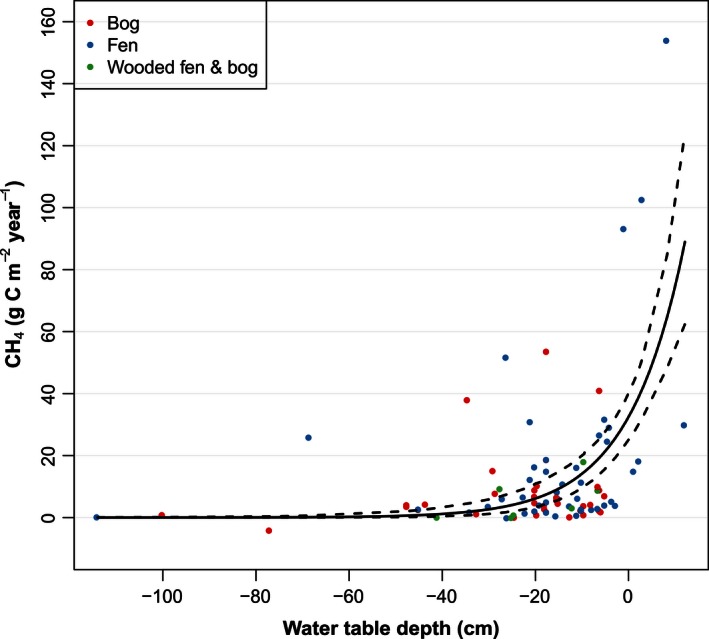
Exponential fitted regression of annual CH_4_ flux and mean annual water level. Methane flux: CH_4_ = 32.462 × exp^(0.08 ×^ ^WT^ ^)^ (*n* = 87, *r* ^2^ = .54, *p* < .01). The dashed lines represent 95% confidence intervals for the regression line

Our results show that natural northern peatland (pristine) sites are important sources for CH_4_ emissions to the atmosphere (Table 1) with an overall average annual flux (mean ± *SD*) covering all sites, vegetation, and locations being 12 ± 21 g C m^−2 ^year^−1^. The median is 4.3 C m^−2 ^year^−1^. However, emissions between the sites were highly variable with a 95% CI of 7.6–15.7 g C m^−2 ^year^−1^ for the mean and 3.3–6.3 g C m^−2 ^year^−1^ for the median. A *t*‐test (*t* = −1.99) shows that CH_4_ emissions from the fen sites (*n* = 59) mean 15.4 g C m^−2 ^year^−1^ are significantly higher (*p* = .05) than those from the bog sites (*n* = 49) 7.1 g C m^−2 ^year^−1^ (Fig. 2). A linear regression between log CH_4_ flux and mean WT depth for different peatland types showed significant correlations for bog (*n* = 87, *r* ^2^ = 0.54, *p* < 0.01) and fen (*n* = 45, *r* ^2^ = 0.13, *p* < 0.01), but not for wooded fen and bog (*n* = 7, *r* ^2^ = 0.36, *p* = 0.09) (Fig. 3a). When four outliers were removed, the correlation was significant for fen (*n* = 43, *r* ^2^ = .22, *p* < .001) but not for bog (*n* = 33, *r* ^2^ = .36, *p* = .8) or wooded fen and bog (*n* = 7, *r* ^2^ = .36, *p* = .09) (Fig. 3B). The significant correlation between log CH_4_ flux and mean WT depth (Fig. 4) suggested an exponential model which was tested by a nonlinear regression and resulted in the following relationship:

$${\text{CH}}_{4}=32.462\;\times \;{\text{exp}}^{\left(0.08\;\times \;\text{WT}\right)}\left(n=87,\;r^{2}=0.54,\;p<0.01\right)$$

**Table 1:** Methane fluxes from natural northern peatlands. MAAT – mean annual air temperature (°C), WT – water table (cm; positive values indicate water depth above the soil surface, and negative values indicate water depth below the soil surface)

Peatland type/location	Coordinates	D (years)	MAAT (°C)	pH^a^	WT (cm)	Annual CH_4_ flux^b^ (g C m^−2^ year^−1^)	References
Bog (FIN)	65°51′N, 30°53′E	2	2.0	3.8–4.6	−15 to (−21)	4.0	Alm, Saarnio, Nykänen, Silvola, and Martikainen (1999)
Fen (FIN)				4.1–5.6	−2 to (−40)		
Bog (Dry; Palsa mire; SWE)	68°22′N, 19°03′E	6	−0.7	ND	ND	0.5	Bäckstrand et al. (2010)
Fen (*Sphagnum angustifolium*; SWE)					(−5) to (−25)	6.2	
Fen (Wet; *Eriophorum* spp.; SWE)					−5.0	31.8	
Bog (DE)	53°41′N, 08°49′E	2	8.5	3.1	−10 to (−80)	4.2	Beetz et al. (2013)
Bog (CA)	45°41′N, 75°52′W	2	6.4	ND	−40 to (−50)	2.7	Brown, Humphreys, Moore, Roulet, and Lafleur (2014)
Open bog (CA)	49°10′N, 82°45′W	1	0.0	4–4.8	ND	0.6	Bubier et al. (1993)
Treed bog (CA)				4.6–4.8	ND	0.5	
Open fen/ dry (CA)				5.4–6.3	21.3–81.7	0.0	
Open fen/ wet (CA)				4.8	12.2–12.9	3.8	
Treed fen (CA)				5.4–6.3	2.7–21.3	3.2	
Fen (GL)	74°30′N, 21°00′W	1	−10.3	ND	0 to (−45)	6.7^c^	Christensen, Friborg, and Sommerkorn (2000)
Bog (USA)	47°32′N, 93°28′W	1	3.0	3.5–7.0	3 to (−43)	9.0	Crill et al. (1988)
Bog (CA)	44°23′N, 65°13′W	2	6.3	ND	11 to (−30)	3.9^c^	Dalva and Moore (2001)
Bog (SL)	45°59′N, 14°30′W	1	10.0	3.2	−24.4	0.2	Danevcic, Mandic‐Mulec, Stres, Stopar, and Hacin (2010)
Bog (USA)	45°94′N, 90°27′W	2	5.7	ND	ND	0.8	Desai et al. (2015)
Bog (hummock; USA)	47°32′N, 93°28′W	2	3.1	ND	−6.1	2.3	Dise, Gorham, and Verry (1993)
Bog (hollow)						9.0	
Junction fen						26.7	
Bog						41.1	
Bog (UK)	55°79′N, 3°24′W	3	10	4.4	−12.5	0.3	Drewer et al. (2010)
Fen (FIN)	67°59′N, 24°12′W	2	−1.4	5.8	1.2	15.0	
Rich fen (CA)	48°21′N, 85°21′W	1	ND	6.3	8.3	154.1^c^	Godin, McLaughlin, Webster, Packalen, and Basiliko (2012)
Intermediate fen (CA)				6.2	3.0	102.7^c^	
Poor fen (CA)				4.8	−22.1	1.5^c^	
Fen (SWE)	64°12′N, 19°34′E	3	1.2	4.0	ND	11.8	Granberg et al. (2001)
Bog (hummock; SWE)	63°44′N, 20°06′E	1	3.3	ND	−19.6	0.9^c^	Granberg et al. (1997)
Bog (lawn; SWE)					−10.1	2.4^c^	
Bog (carpet; SWE)					−05.8	1.9^c^	
Poor fen (SWE)					−15.2	8.4^c^	
Sedge fen (SWE)	64°20′N, 18°18′E	1		ND	−2.7	4.0^c^	
Poor fen (SWE)	64°24′N, 20°11′E	1		ND	−3.5	5.3^c^	
Poor fen (SWE)	63°44′N, 20°02′E	1		ND	−7.8	2.7^c^	
Bog (SWE)	63°36′N, 19°37′E	1		ND	−9.5	1.0^c^	
Poor fen (SWE)	64°02′N, 20°40′E	1		ND	−15.5	0.6^c^	
Fen (CA)	58°39′N, 93°49′W	4	3.0	ND	−15 to 20	5.1	Hanis, Tenuta, Amiro, and Papakyriakou (2013)
Fen (FIN)	69°14′N, 27°17′E	3	0.4	4.5	0 to (−10)	4.1	Hargreaves, Fowler, Pitcairn, and Aurela (2001)
Fen (treed fen; FIN)	67°00′N, 27°00′E	2	−1.0	ND	−15 to 4	18.1	Huttunen et al. (2003)
Fen (FIN)					−1 to 21	16.3	
Eutrophic fens (FIN)					−26 to 2	11.0	
Fen (spruce mires; FIN)					−37 to (−13)	0.1	
Bog (SWE)	68°20′ N, 19°03′E	2	−0.9	ND	ND	20.3	Jackowicz‐Korczynski et al. (2010)
Fen (FIN)	60°26′ N, 23°38′E	1	ND	4.6–4.7	2.3	18.3^c^	Juottonen et al. (2012)
	62°16′ N, 23°48′E	1	ND	4.9–5.1	−0.9	93.3^c^	
	64°04′ N, 26°40′E	1	ND	5.1–5.3	12.1	30^c^	
Fen (PL)	52°45′ N, 16°18′E	2	6.8	6.2	−4.0	29.2	Juszczak and Augustin (2013)
Bog (CA)	45°41′N, 75°52′W	2	6.0	ND	−19 to (−38.1)	7.9	Lai, Moore, and Roulet (2014)
Blanket bog (IRE)	51°55′N, 9°55′W	3	10.5	4.4–4.7	5 to (−25)	4.7	Laine, Wilson, Kiely, and Byrne (2007)
Open graminoid bog (CA)	61°08′N, 121°04′W	0.2	−3.7	ND	−5 to (−35)	4.9^c^	Liblik et al. (1997)
Open graminoid fen					−4 to (−9)	3.0^c^	
Open graminoid poor fen					−8 to (−14)	8.0^c^	
Open fen (low shrub)					−14 to (−35)	0.9^c^	
Fen (tree/low shrub)					−39 to (−43)	0.2^c^	
Bog (tree low/tall shrub)					ND	0.0^c^	
Fen (CA)	54°95′N, 112°46′W	1	2.1	ND	−30 to (−60)	2.8^c^	Long, Flanagan, and Cai (2010)
Bog (SWE)	56°15′N, 13°33′E	1	6.2	ND	0 to (−16)	4.3^c^	Lund et al. (2009)
Bog (SWE)	62°20′N, 18°58′E	1	−0.8	ND	ND	1.5^c^	
Raised bog (EE)	58°34′N, 24°23′E	1	ND	4.2	ND	1.8	Mander et al. (2012)
Fen (meadow; EE)						1.1	
Bog (hummock; CA)	45°41′N, 75°48′W	5	6.0	ND	−35 to (−52)	4.4^c^	Moore et al. (2011)
Bog (lawn)					−27 to (−31)		
Bog (*Eriophorum vaginatum*)					−23 to (−46)		
Fen (hummock; SWE)	MS	1	5.0	ND	−30.0	3.7	Nilsson et al. (2001)
Fen (transitional fens)					−34.0	1.9	
Fen (low sedge fens)					−27.0	6.2	
Fen (tall sedge fens)	64°18′N, 19°33′E				−21.0	12.4	
Poor fen (SWE)	62°45′N, 31°03′E	2	1.2	4.3 to 5.3	0 to (−20)	11.5	Nilsson et al. (2008)
Fen (FIN)	MS	2	1.9	5.3	−20 to (−117)	26.0	Nykänen, Alm, Lang, Silvola, and Martikainen (1995)
Bog (FIN)	MS	2	2.5	3.7 to 4.3	−1.1 to (−39)	6.9	Nykänen et al. (1998)
Fen	69°49′N, 27°10′E			4.4–5.6		16.4	
Fen (wet; FIN)		2	−1.2	ND	−4.2 to (−4.6)	24.7	Nykänen, Heikkinen, Pirinen, Tiilikainen, and Martikainen (2003)
Bog (dry; FIN)	68°22′N, 19°03′E					1.0	
Bog (SWE)	57°00′N, 82°00′E	2	−0.5	ND	0 to (−35)	1.9	Olefeldt et al. (2012)
Bog (RU)	53°54′N, 78°46′W	5	ND	ND	ND	19.4	Panikov and Dedysh (2000)
Rich fen (CA)	53°38′N, 77°43′W	1	−3.1	ND	−8 to (−30)	4.1^c^	Pelletier, Moore, Roulet, Garneau, and Beaulieu‐Audy (2007)
Raised bog	53°34′N, 76°08′W				−6.7 to (−29)	2.9	
Fen (hummock; shrubs)	46°19′N, 86°03′W		−16.6			4.9^c^	
Poor fen (USA)	61°50′N, 24°12′E	1	5.0	3.8	−5 to (−30)	15^c^	Pypker et al. (2013)
Boreal fen (FIN)	45°04′N, 78°45′W	2	3.3	ND	−5 to (−50)	9.4	Rinne et al. (2007)
Bog (CA)		1	4.4	4.3–5.5	−29 to (−36)	1.3	Roulet, Ash & Moore (1992)
Fen	50°30′N, 80°23′W			4.8	−114.0	0.3	
Treed fen (shrubs; CA)	64°18′N, 19°33′E &51°35′N, 81°48′W	1	−1.2	ND	ND	0.3	Roulet et al. (1994)
Open fen						0.5	
Open bog						3.5	
Rich bog (shrub)						3.0	
Treed bog						0.1	
Fen (conifer forest)						0.1	
Open fen (CA)	58°45′N, 94°09′W	1	−7.2	ND		5.0	
Treed bog						0.0	
Raised bog (CA)	45°41′N, 75°48′W	6	6.0	3	−20 to (−75)	3.7	Roulet et al. (2007)
Bog (USA)	42°27′N, 84°01′W	3	ND	4.2	−50 to 15	53.7	Shannon and White (1994)
Bog (USA)	58°45′N, 94°09′W		3.9		−50 to 15	18.8	Shannon and White (1994)
Fen (hummock; CA)	46°40′N, 71°10′W	2	ND	ND	−14 to (−21)	1.8	Strack et al. (2004)
Fen (lawn; CA)		2	ND	ND	−6 to (−14)	2.8	
Fen (hollow; CA)		2	ND	ND	0 to (−20)	2.2	
Treed bog (CA)	47°96′N, 69°42′W	1	5.2	ND	−15.3	6.6	Strack & Zuback (2013)
Boreal fen (USA)	53°57′N, 105°57′W	1	ND	7.1	−5 to (30)	17.7^c^	Suyker, Verma, Clement, and Billesbach (1996)
Fen (SWE)	ND	2	−0.7	ND	ND	20.2	Tang et al. (2015)
						22.6^M^	
Poor fen (USA)	43°12.5′N, 71°3.5′W	5	8.1	4.1–5.7	9.4 to 29.9	31.0^c^	Treat et al. (2007)
Fen (CA)	54°06′N, 72°30′W	2	−4.3	ND	−5.4 to (−16.3)	6.3	Trudeau, Garneau, and Pelletier (2013)
Rich fen (USA)	64°82′N, 147°87′W	2	−2.9	5.3	ND	2.8^c^	Turetsky et al. (2008)
Fen (CZ)	49°09′N, 13°22′E	3	4.0	ND	−7.2 to (−45.2)	51.8^c^	Urbanova, Barta, and Picek (2013)
Bog (CZ)	48°58′N, 13°27′E	3	3.2	ND	−2.9 to (−36.1)	10.4^c^	
Bog (low shrub; CZ)	48°58′N, 13°27′E	2	3.2	ND	−2.3 to (−10.6)	8.9^c^	Urbanova, Picek, and Tuittila (2013)
Bog (*Trichophorum* lawn)				ND		10.1^c^	
Eccentric bog (SWE)	63°44′N, 20°06′E	2	ND	ND	−30 to 124	4.0	Waddington and Roulet (2000)
Fen (RU)	72°22′N, 126°30′E	1	−14.7	ND	−10.0	2.4	Wille, Kutzbach, Sachs, Wagner, and Pfeiffer (2008)
Bog (UK)	MS	1	5.8	6.0	−20.0	7.1	Worrall, Reed, Warburton, and Burt (2003)
Bog (UK)	54°09′N, 04°11′W	2	9.4	3.6	−15.3	5.8	Yamulki et al. (2013)

D, duration (years); ND, no data; M, modeled; MS, multiple sites; CA, Canada; CZ, Czech Republic; EE, Estonia; FIN, Finland; DE, Germany; GL, Greenland; IRE, Ireland; PL, Poland; RU, Russia; SL, Slovenia; SWE, Sweden; UK, United Kingdom; and USA, United States of America.

a Different methods were used to measure soil pH using pH probe/meter in deionized water or 0.01 M CaCl_2_ in 1:1 and 1:2, or 1:5 (v:v) soils: solution ratios.

b Average values were measured/calculated and converted to g C m^−2^ year^−1^ using original data.

c Annual values were estimated from the original seasonal measured values. Methane gas flux during winter was considered as 15% from the annual flux following the suggestions of Saarnio et al. (2007) and Maljanen et al. (2010).

[Correction added on 24 September 2016, equations in the Results section were incorrect and have been corrected in this version.]

**Figure 5:**
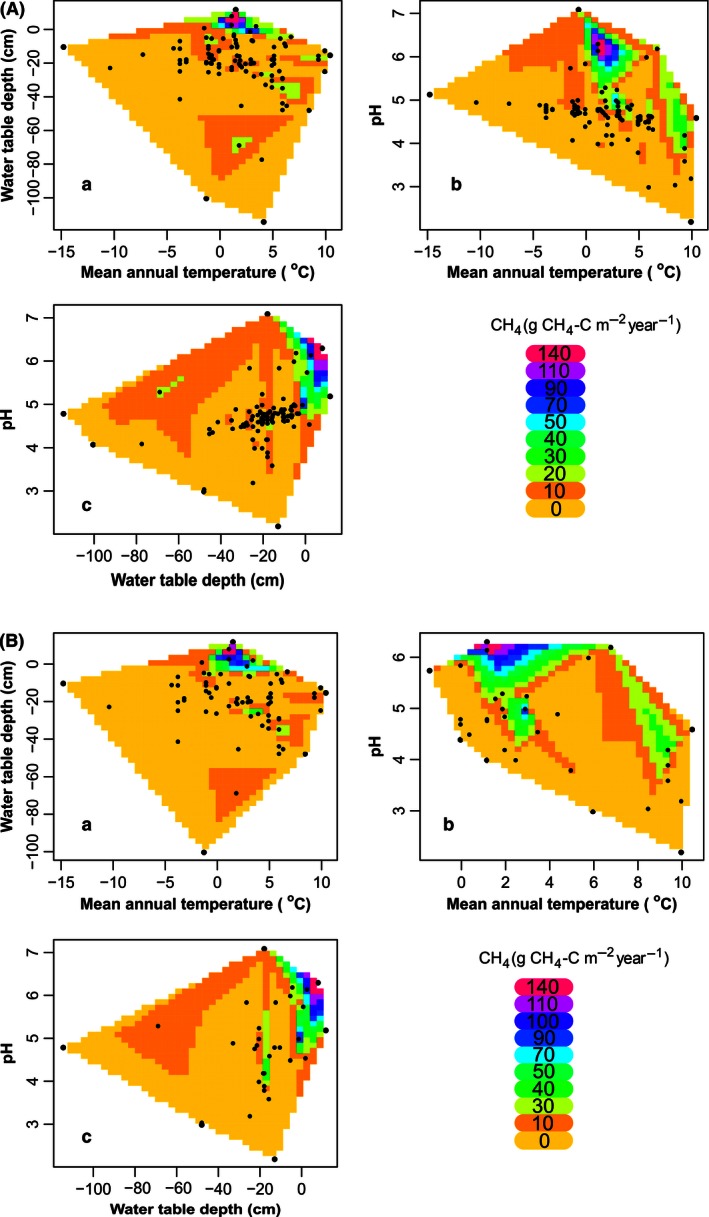
Contour plots of imputed data showing relationships between the annual CH_4_ flux and environmental parameters: (A) when only available data used: (a) mean annual temperature and mean water table below the surface (*n* = 76). These two variables explain 8.5% of CH_4_ flux overall variation (*p* < .05); (b) mean annual temperature and soil pH (*n* = 33). These two variables explain 16.3% of CH_4_ flux overall variation (*p* < .05); (c) soil pH and mean water table below the surface (*n* = 32). These two variables explain 17.8% of CH_4_ flux overall variation (*p* < .05). (B) when data were imputed (*n* = 108): (a) mean annual temperature and mean water table below the surface. These two variables explain 7.6% of CH_4_ flux overall variation (*p* < .01); (b) mean annual temperature and soil pH. These two variables explain 19.7% of CH_4_ flux overall variation (*p* < .001); (c) soil pH and mean water table below the surface. These two variables explain 16.0% of CH_4_ flux overall variation (*p* < .05)

The contour plots in Fig. 5A show a trend toward higher CH_4_ emissions with a high water table and high pH and with lower temperature, peaking at a mean annual air temperature around 2°C. The LMM results with samples that had a pH value (30 observations/samples) showed that pH is a statistically significant factor (*p* = .04). The proportion of variance explained by the fixed factor(s) alone is 34% of CH_4_ flux variation. The proportion of variance explained by both the fixed and random factors is 53%. The LMM results with samples in cases when the variable “pH” was omitted, where the number of observations is 76, shows that “peatland type” and WT are statistically important factors (*p* < .05 & *p* < .01, respectively). The proportion of variance explained by the fixed factor(s) alone is 19% of CH_4_ flux variation. The proportion of variance explained by both the fixed and random factors is 42%.

When missing data values are imputed using missMDA, the contour plots in Fig. 5B show a similar pattern to those of the raw data in Fig. 5A, validating the technique. When LMM analysis is made on the imputed data with 108 observations, it shows that peatland type (*p* < .05), pH (*p* < .001), WT (*p* < .001), and air temperature (*p* < .01) are statistically important factors in determining CH_4_ flux. The proportion of variance in CH_4_ flux explained by the fixed factor(s) alone is 31%. The proportion of variance explained by both the fixed and random factors is 63% (Table 2).

**Table 2:** Relationships between annual CH_4_ flux and environmental variables (WT and pH) and type of peatland using linear mixed‐effects model (LMM)

	Estimates	*SE*	*df*	*T* value	*p*‐value^a^
Intercept	0.387	0.562	100	0.602	.55
Peatland type	0.271	0.122	91	2.224	.03*
pH	0.465	0.112	102	4.134	7.32e^−5^***
Water table	0.125	0.004	103	3.549	.58e^−3^***
Air temperature	0.514	0.019	80	2.687	.88e^−2^**

Missing values were imputed using missMDA software. This produced 108 observations for LMM analysis. Peatland type, pH, WT, and air temperature are statistically important factors in this case. The proportion of variance explained by the fixed factor(s) alone is 31% of CH_4_ flux variation. The proportion of variance explained by both the fixed and random factors is 63%.

a Significant codes: 0 = ***; 0.001 = **; 0.01 = *.

### 3.2 Methane emissions from drained peatlands

**Figure 6:**
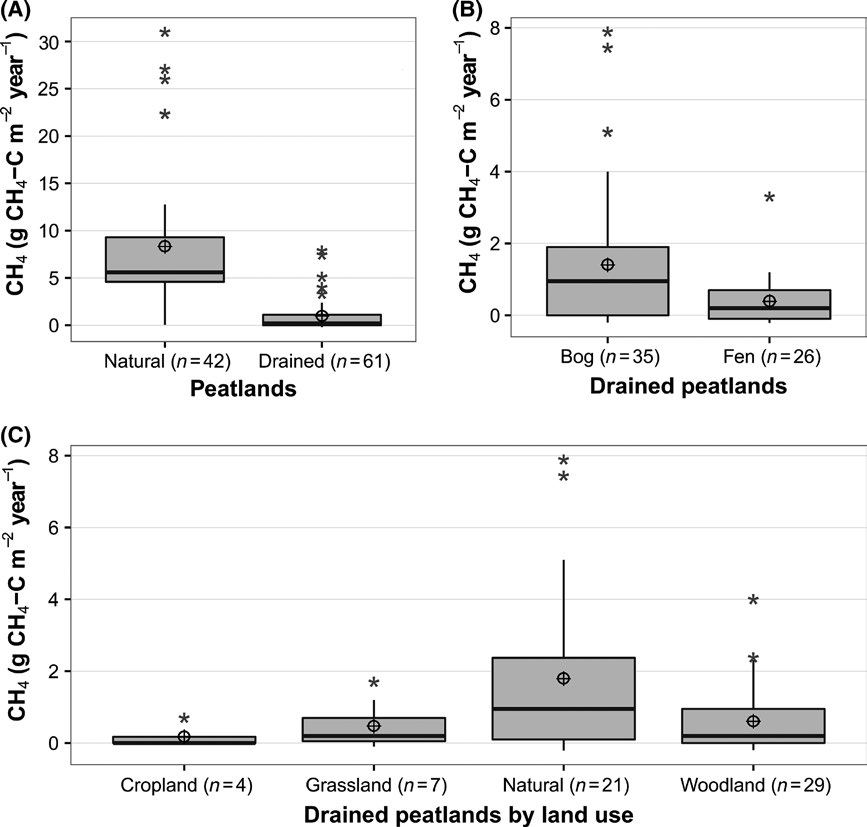
Effects of drainage on CH_4_ emissions from peatlands. Box and whiskers plots showing median, 25 and 75% median quartiles, mean (⊕), 95% confidence interval (whiskers), and outlier values (*). (A) Comparison of annual CH_4_ flux from drained and natural peatlands. *T*‐test indicates a significant difference (*t* = 7.25; *p* < .001) between the two groups, (B) CH_4_ flux of drained peatland by type, bog and fen, and *t*‐test indicates a significant difference between bog and fen (*t* = 2.46; *p* < .05). (C) CH_4_ flux of drained peatland by land use: cropland, grassland, natural, and woodland/shrubs; ANOVA shows significant differences between the four groups (*F* = 2.98; *p* < .05)

A *t*‐test shows that the difference in CH_4_ emissions between drained (*n* = 61) and natural peatlands (*n* = 42) is significant (*t* = 7.25, *p* < 0.001) (Fig. 6a). Drainage reduced the CH_4_ flux by, on average, 84% compared to the original emission values with a mean of 8.3 g C m^−2^ year^−1^. Drainage reduced CH_4_ emissions from the fen ecosystems by more than that from bog ecosystems, and a *t*‐test showed a significant difference (*t* = 2.46, *p* < .015) between fens and bogs (Fig. 6B). This effect is similar for all types of drained peatland regardless of the land use and vegetation cover. A paired *t*‐test to assess the effect of drainage for paired sites of bogs and fens showed that for the bogs (*t* = 4,443; *p* < 0.001; *n* = 25) and for the fens (*t* = 3,762; *p* < 0.01; *n* = 17). A one‐way ANOVA shows that the difference in CH_4_ emissions after drainage between the land use/land cover of crops (*n* = 4), grass (*n* = 7), natural (*n* = 21), or woodland (*n* = 29) is significant (*F* = 2.98, *p* < 0.05) (Fig. 6c).

### 3.3 Methane emissions from restored peatlands

**Figure 7:**
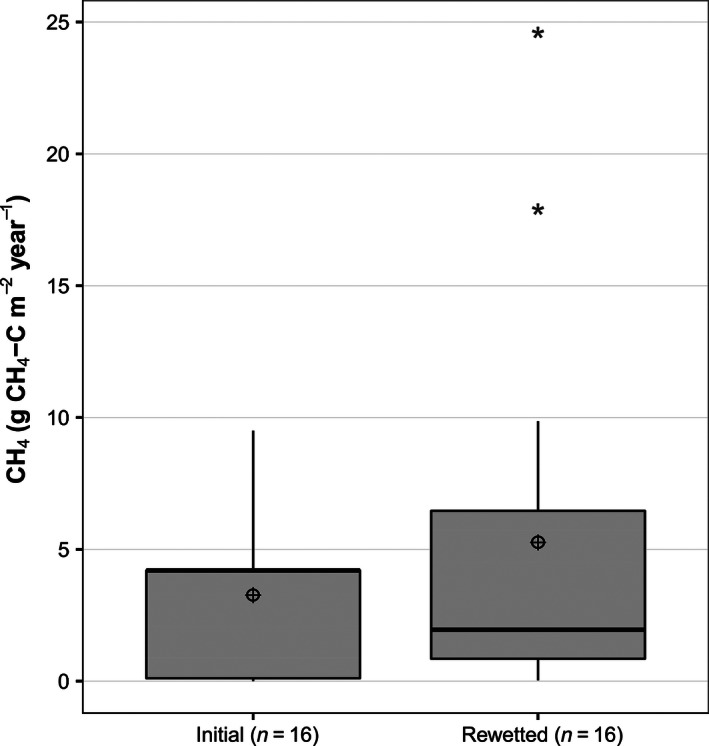
Effects of rewetting on annual CH_4_ flux from peatlands. Box and whiskers plots showing median, 25 and 75% median quartiles, mean (⊕), 95% confidence interval (whiskers), and outlier values (*). The left box shows initial methane flux with anthropogenic drained land management, whereas the right one indicates CH_4_ flux after restoring vegetation and/or rewetting

Only 16 sites explicitly measured the effect of rewetting peatlands that had previously been drained for many uses, including forestry, cropping grazing, and mining. There were insufficient data for each category of initial land use, but considering the entire dataset (*n* = 16) rewetting increased methane flux by an average of 1.3 ± 6.5 g C m^−2^ year^−1^ (46%). However, a paired *t*‐test showed that the change in CH_4_ flux due to rewetting was not statistically significant with mean flux before restoration being 3.0 ± 3.1 g C m^−2^ year^−1^ and after restoration being 4.2 ± 6.3 g C m^−2^ year^−1^ (*p* = .37) with a pooled standard deviation of 6.0 (Fig. 7).

This indicates a different response to rewetting between sites, which all have different previous anthropogenic management, land use, and initial peatland type. The published data are insufficient to identify why CH_4_ emissions from the different sites respond differently after rewetting.

## 4 Discussion

### 4.1 Methane emissions from northern natural peatlands

This review and meta‐analysis shows that natural northern peatlands are a significant source for CH_4_ emissions to the atmosphere due to prevailing waterlogged conditions (Huttunen, Nykänen, Turunen, & Martikainen, 2003). This is in agreement with other previous studies carried out by Nilsson et al. (2001), Christensen et al. (2003), Zhuang et al. (2006), Lai (2009) and Turetsky et al. (2014). However, high variability was observed between the sites with 95% CI of 7.6–15.7 g C m^−2 ^year^−1^ for the mean and 3.3–6.3 g C m^−2 ^year^−1^ for the median, especially on flooded peatlands (Couwenberg & Fritz, 2012). The type and composition of dominant peatland vegetation (Bubier, 1995; Turetsky et al., 2014) influence CH_4_ emission dynamics, both by adding labile C substrates for CH_4_ production (Ström, Ekberg, Mastepanov, & Christensen, 2003) and by maintaining gas conduits, which affect the production, oxidation, and transportation of CH_4_ (Joabsson et al., 1999). Bogs and fens differ in biotic and abiotic factors. These biotic and abiotic differences lead to the fens having the highest methanogenic activity (Juottonen et al., 2005), highest litter degradation rate (Aerts, Verhoeven, & Whigham, 1999), and thereby highest CH_4_ emissions (Nykänen et al., 1998), compared to the bogs. However, both fen and bog ecosystems (Granberg et al., 1997; Lund, Christensen, Mastepanov, Lindroth, & Strom, 2009; Nilson et al., 2001; Rinne et al., 2007) are sources for CH_4_ emissions which may cause these peatland types to be a net GHG source to the atmosphere (Drewer et al., 2010). The microtopography of a peatland is not uniform, with many hummocks and hollows, which can result in highly variable CH_4_ emissions from the same site (Lai, 2009). Differences in methane emissions between the hummocks and hollows could be explained by the higher CH_4_ oxidation in the thicker aerobic acrotelm layers of the hummocks (Waddington and Roulet, 1996) and the higher CH_4_ productions in the hollows due to high WT and temperature (Bubier et al., 1993).

A number of dynamic biological processes control CH_4_ emissions from northern peatlands. However, gas production and consumption are mainly due to methanogenic and methanotrophic microbiota, respectively. Methane transport to the atmosphere takes place either physically (by diffusion and ebullition) or biologically (by a plant‐mediated process) (Lai, 2009). Our analysis suggests that the emission of CH_4_ from northern peatlands is mainly controlled by WT depth (Granberg et al., 1997; Moore & Knowles, 1989), plant community composition (Granberg et al., 1997; Nilsson et al., 2001), and soil pH (Hutsch, 1998; Singh et al., 1999). Nevertheless, the influence of soil pH on CH_4_ emissions is uncertain because laboratory‐measured soil pH may differ from field pH. Our analysis shows that the optimal WT for CH_4_ emissions was consistently below the peat surface in the bogs and near to the peat surface for the fens. A similar conclusion was also reported by Turetsky et al. (2014). Many studies have reported the influence of WT depth (Frenzel & Karofeld, 2000; Granberg et al., 1997; Moore & Dalva, 2006; Yang et al., 2006), pH (Hutsch, 1998; Singh et al., 1999), and temperature (Ding & Cai, 2007; Granberg et al., 1997; Saarnio et al., 1998) on CH_4_ emissions. Deep WT can reduce CH_4_ emissions from peatlands (Strack, Waddington, & Tuittila, 2004), but it may encourage the domination of vascular plant species over mosses which can increase CH_4_ production (Bellisario, Bubier, & Moore, 1999). In this review, however, mean annual air temperature is not a strong predictor for CH_4_ emissions, and the interaction between mean annual air temperature, plant community composition, and soil WT depth is important (Granberg et al., 1997) [e.g., a clear relationship of CH_4_ emissions on soil temperature at certain WT depth reported by Nadeau, Rousseau, Coursole, Margolis, and Parlange (2013) and Olson, Griffis, Noormets, Kolka, and Chen (2013)]. Here, we observe that CH_4_ emissions are highest at a MAAT around 2°C, decreasing above and below that value.

The response of CH_4_ emissions in peatlands to temperature appears to be somewhat unpredictable. Most of the studies report a clear dependence of CH_4_ emission intensity on the soil temperature (Christensen et al., 2003; Gedney, Cox, & Huntingford, 2004; Mastepanov et al., 2013; Treat et al., 2007; Updegraff, Bridgham, Pastor, Weishampel, & Harth, 2001). Likewise, models of CH_4_ emission consider soil temperature as a main driver (Bridgham, Cadillo‐Quiroz, Keller, & Zhuang, 2013; Walter & Heimann, 2000). However, a combined chamber and eddy covariance study by Pypker, Moore, Waddington, Hribljan, and Chimner (2013) shows that daily mean soil temperature at 20 cm depth was poorly correlated with changes in CH_4_ (17%) when the ecosystem represented a net CO_2_ sink (negative net ecosystem exchange, NEE), but the correlation increased to 34% when it was a net CO_2_ source (positive NEE). This indicates shifting temperature controls on the CH_4_ flux throughout the growing season (Treat et al., 2007).

Natural northern peatlands have an important impact on climate change (Christensen et al., 2003; Lai, 2009; Nilsson et al., 2001; Turetsky et al., 2014), and climate change has an impact on northern peatlands. In the Nordic region, under climate change, temperature is predicted to increase and WT to decrease (Forster et al., 2007). Temperature may accelerate changes in soil microbial processes, vegetation dynamics, and chemistry of pore water, all of which will affect CH_4_ cycling (Weltzin, Bridgham, Pastor, Chen, & Harth, 2003; White, Shannon, Weltzin, Pastor, & Bridgham, 2008). High temperature will also result in melting of the permafrost and release of CH_4_ to the atmosphere, which may provide a positive feedback to climate change in the short term (Friedlingstein et al., 2006; Olefeldt, Turetsky, Crill, & McGuire, 2013). The larger unsaturated zone will lead to less CH_4_ emissions, and some dry sites may become sinks for CH_4_ (Worrall, Burt, & Adamson, 2006). McCalley et al. (2014) reported that microbial community response to permafrost thaw will regulate CH_4_ dynamics. However, the majority of models forecast a significant warming‐related decrease of CH_4_ emissions from northern peatlands (Bridgham et al., 2013; Frolking et al., 2011). In most temperate wetlands, over the long term (300 years), C sequestration is expected to compensate for the warming role of CH_4_, turning most wetlands to net C sinks with net negative radiative forcing (Mitsch et al., 2013).

### 4.2 Methane emissions from drained peatlands

The drainage practices in northern peatlands clearly reduce CH_4_ emissions under all types of land use and vegetation, on average by 84% (Table 3). Drainage practices improve aeration (Schrier‐Uijl, Veenendaal, Leffelaar, van Huissteden, & Berendse, 2010) leading to lower CH_4_ emissions. They decrease C input, from decomposing plants, to the methanogenic anaerobic layer (Basiliko, Yavitt, Dees, & Merkel, 2003; Bergman, Svensson, & Nilsson, 1998; Bergman et al., 2000). Drainage also increases CH_4_ oxidation to CO_2_ and thereby reduces CH_4_ emissions (Holden, 2005; Moore & Dalva, 2006; Sundh, Nilsson, Mikkela, Granberg, & Svensson, 2000). Moreover, Yrjälä et al. (2011) found that several years of drying changed the structure of the plant community and thereby microbial communities that control functions of GHG emissions. Similar results of decrease in CH_4_ emissions under drainage were reported by Bussell, Jones, Healey, and Pullin (2012) and Turetsky et al. (2014). However, drainage influences CH_4_ emissions from fens more than from bogs. This is because WT depth in the fen sites is more sensitive to drainage compared to the bog sites (Maljanen et al., 2010).

**Table 3:** Methane fluxes from drained peatlands

Peatland type/location	Coordinates	D (years)	Trophic level/vegetation	CH_4_ flux^a^ (g C m^−2^ year^−1^)		% change	References
				Natural	Drained		
Bog (FIN)	MS	2	Ombrotrophic	4.0	2.1	−48	Alm et al. (1999)
Fen (FIN)	MS	2	Oligotrophic	31.0	0.0	−100	
Fen (DE)	52°30′N, 08°20′E	4	Cropland	ND	0.7		Beyer, Liebersbach, and Höper (2015)
Fen (DE)			Grassland	ND	−0.1		
Bog (EE)	58°52′N, 26°14′E	2	Ombrotrophic	2.7	0.9	−66	Carter, Sutton, and Stenglen (2012)
Blanket bog (UK)	52°58′N, 03°49′W	2.3	*Eriophorum vaginatum*;* Sphagnum* spp.	4.5	3.3	−27	Cooper et al. (2014)
Bog (forest; SL)	45°58′N, 14°28′E	1	*Betula spp., Frangula alnus*	0.2	−0.2	>−100	Danevcic et al. (2010)
Fen (SL)	45°58′N, 14°28′E	1	Grassland; WT = −53.2 cm	ND	0.2	n/a	
Fen (SL)		1	Grassland; WT = −96.7 cm	ND	0.2	n/a	
Bog (UK)	55°47′N, 3°14′W	2	Patchy mix of grasses, sedges & soft rush	ND	0.1	n/a	Dinsmore, Skiba, Billett, and Rees (2009)
Bog (cropland; CA)	45°08′N, 73°26′E	1	Onion	ND	0.0	n/a	Glenn, Heyes, and Moore (1993)
			Celery		0.0	n/a	
			Occasional shrubs/ herb		0.0	n/a	
Bog (forest; CA)			Trees/ shrub/ herb		0.0	n/a	
			Trees/ shrub/ herb		0.0	n/a	
Bog (cropland; CA)	45°09′N, 73°40′E	1	Celery	ND	0.0	n/a	Glenn et al. (1993)
			Grass		0.0	n/a	
Bog (forest; CA)			Trees/ shrub/ herb		0.0	n/a	
Bog (FIN)	60°38′N, 24°21′E	1.3	Dwarf shrub pine	ND	−0.1	n/a	Lohila et al. (2011)
Fen (cropland; FIN)	MS	5	Birch–pine–alder	ND	−0.1	n/a	Mäkiranta et al. (2007)
Fen (cutaway peat)			Birch–pine		0.0	n/a	
Bog (afforested; FIN)	64°06′N, 24°21′E	2	Birch; 1 year old	ND	1.0^b^	n/a	Maljanen, Hytönen, and Martikainen (2001)
Bog (afforested)			Pine; 6 years old		0.7^b^	n/a	
Bog (afforested)			Pine; 23 years old		−0.1^b^	n/a	
Fen (FIN)	MS	2	*Eriophorum angustifolium*	5.6	0.2	−96	Minkkinen and Laine (2006)
			*E. vaginatum*		−0.1	>−100	
			*Sphagnum sp*.		−0.1	>−100	
			Forest moss		−0.2	>−100	
			Litter		−0.1	>−100	
Bog (FIN)	MS	2	*E. vaginatum*	5.0	5.1	2	
			*Sphagnum angustifolium*		1.4	−71	
			Forest moss		0.4	−93	
Fen (forest; FIN)		3	Mesotrophic treed	0.1	0.0	−100	Minkkinen, Korhonen, Savolainen, and Laine (2002)
			Mesotrophic treeless	0.1	0.0	−100	
			Mesotrophic sparsely treed	9.0	1.1	−88	
			Oligotrophic treed	22.3	1.0	−96	
			Oligotrophic treeless	4.9	1.1	−77	
			Oligotrophic sparsely treed	22.3	1.0	−96	
			Ombrotrophic treed	22.3	1.0	−96	
Raised bog (FIN)			Ombrotrophic treed	5.4	1.2	−77	
			Ombrotrophic treeless	11.7	7.4	−36	
			Ombrotrophic sparsely treed	4.9	2.3	−53	
Blanket bog (forest; UK)	55°10′N, 02°03′W	2	Sitka spruce	1.3	0.5	−65	Mojeremane, Rees, and Mencuccini (2010)
Fen (FIN)	62°45′N, 31°03′E & 62°40′N, 30°50′E	2	Virgin fen	26.0	0.1	−100	Nykänen et al. (1995)
Bog (FIN)	62°45′N, 31°03′E &62°40′N, 30°50′E	2	Ombrogenous bog	13.0	7.9	−38	Nykänen (1998)
			Ombrogenous pine forest	5.3	2.4	−55	
			Dwarf shrub pine bush	5.9	1.1	−81	
			Minerogenous oligotrophic	27.1	−0.2	>−100	
			Minerogenous mesotrophic	1.0	0.9	−4.4	
Bog (EE)	MS	1	Ombrotrophic	8.5	2.4	−72	Salm et al. (2012)
Fen (CA)	46°40′N, 71°10′W	2	Hummocks	1.8	0.2	−89	Strack et al. (2004)
			Lawns	2.8	1.2	−57	
			Hollows	2.2	3.3	50	
Fen (USA)	64°82′N, 147°87′W	2	Rich fen/ Warm	2.8^b^	1.8^b^	−36	Turetsky et al. (2008)
			Rich fen/ unwarm	2.2^b^	1.3^b^	−41	
Bog (CZ)	49°10′N, 13°19′E	2	High shrubs	10.8	0.2^b^	−98	Urbanova, Barta et al. (2013)
		2	*Molinia caerulea*	9.4	1.7^b^	−82	
		2	*M. caerulea; Calluna vulgaris; E. vaginatum & Vaccinium uliginosum*	9.4	4.0^b^	−57	
Fen (forest; SWE)	57°8′N, 14°45′E	2	Black alder	5.7	0.7	−88	Von Arnold, Nilsson et al. (2005)
		2	Downy birch		0.7	−88	
Fen (forest; SWE)	57°8′N, 14°45′E	2.5	Norway spruce (young trees)	8.6	0.0	−100	Von Arnold, Weslien et al. (2005)
			Norway spruce (old trees)		0.2	−98	
			Pine		0.8	−91	

D, duration (years); ND, no data; MS, multiple sites; WT, water table (cm; positive values indicate water depth above the soil surface, and negative values indicate water depth below the soil surface). n/a, not applicable; CA, Canada; CZ, Czech Republic; EE, Estonia; FIN, Finland; DE, Germany; SL, Slovenia; SWE, Sweden; and UK, United Kingdom.

a Average values were measured/calculated and converted to g C m^−2^ year^−1^ using original data. A negative value indicates CH_4_ uptake, and a positive value indicates CH_4_ emission.

b Annual values were estimated from the original seasonal measured values. Methane flux during winter was considered as 15% from the annual flux following the suggestions of Saarnio et al. (2007) and Maljanen et al. (2010).

Drainage ditches themselves can become new anaerobic zones, with similar characteristics to the undrained peat, with similar or even increased CH_4_ emissions (Huttunen et al., 2003; Schrier‐Uijl et al., 2010; Sundh et al., 2000). In fen meadows in the Netherlands, Schrier‐Uijl et al. (2010) found that ditches and bordering edges contributed up to 60–70% of the total farms’ CH_4_ emissions. These higher emissions from drainage ditches could be large enough to compensate for the reduced CH_4_ emissions by drainage on the remainder of the drained peatland area (Minkkinen, Byrne, & Trettin, 2008). In contrast, Minkkinen, Laine, Nykänen, and Martikainen (1997) reported that CH_4_ emissions from ditches in a drained peatland plantation in Finland during the summer represent about only 4.5% of CH_4_ emissions. Sundh et al. (2000) found that CH_4_ emissions from harvested and drained peat can be kept lower than that from virgin peatland by keeping the ditches clear and free from vegetation.

Drainage and cultivation result in significant reductions in CH_4_ emissions, although it may increase other GHG emissions, that is, CO_2_ and N_2_O (Oleszczuk, Regina, Szajdak, Höper, & Maryga−nova, 2008). The microbial production of CH_4_ is anaerobic, while the production of CO_2_ is aerobic. Therefore, the production and consumption of these two greenhouse gases in peat soils are highly dependent on the oxygen availability in the soil and, thus, the depth of the water table (Aerts & Ludwig, 1997). In fen and bog peatlands, drainage decreased CH_4_ emission but increased CO_2_ emission by more than one order of magnitude (Von Arnold, Nilsson et al., 2005; Von Arnold, Weslien et al., 2005; Yamulki, Anderson, Peace, and Morison (2013). This reduction in CH_4_ emissions, in association with the primary productivity of vegetation, could decrease the total climate forcing of peatlands over the coming century (Worrall et al., 2011). There is a probability of 69% that drainage will result in an overall improvement in the GHG budget due to less CH_4_ emissions (Worrall et al., 2011). Nevertheless, the timescale over which this GHG budget is calculated has an influence, since loss of CO_2_ after drainage can be very long‐lasting (Maljanen et al., 2010). In contrast, Oleszczuk et al. (2008) noted that drainage could increase CO_2_ emissions, with CO_2_ having a longer atmospheric lifetime relative to CH_4_, so the loss of C and lower C sink capacity in drained peatlands could result in increased climate forcing over time. This uncertainty in CH_4_ changes over time is due to the limited long‐term (>10 years) studies on drained northern peatlands.

### 4.3 Methane emissions from restored peatlands

Restoration of drained northern peatlands by rewetting increased CH_4_ emissions compared to the original prewetting emission. In this meta‐analysis, restoration increased CH_4_ flux by 46% (Table 4). Here, the open water pools behind ditch blocks increase the gas emissions (Baird, Holden, & Chapman, 2009). Hahn‐Schofl et al. (2011) reported significantly higher CH_4_ emissions from flooded fen grasslands in Germany, because of high availability of fresh organic matter. Methane emission could be reduced by creating different vegetation compositions (Komulainen, Nykänen, Martikainen, & Laine, 1998; Tuittila et al., 2000; Waddington & Day, 2007) that lead to changes in the methanogenic community and peat properties (Basiliko, Knowles, & Moore, 2004). Mahmood and Strack (2011) reported a significant correlation between CH_4_ emissions and vegetation cover on an abandoned peatland. This is because vegetation stimulates CH_4_ emissions by providing substrates for gas production and transportation to the atmosphere (Wilson, Farrell, Muller, Hepp, & Renou‐Wilson, 2013). In Canada and Ireland, CH_4_ emissions from restored cutover peatlands increased in the first 3 years following restoration due to the fresh substrates provided by the new vegetation cover (Waddington & Day, 2007; Wilson et al., 2013). Fast decomposing litter following restoration of a bog peat could result in higher CH_4_ flux, which could dominate GHG emissions up to 30 years following rewetting (Vanselow‐Algan et al., 2015).

**Table 4:** Effects of restoration on CH_4_ fluxes from peatlands

Peatland type/location	Coordinates	D (years)	Type of management/vegetation	CH_4_ flux^a^ (g C m^−2^ year^−1^)		% change	References
				Natural	Restored		
Bog (DE)	53°41′N, 8°49′E	2	Rewetted (intensive grassland)	4.2	0.1	−97	Beetz et al. (2013)
		2	Rewetted (extensive grassland)	4.2	0.9	−79	
Bog (DE)	53°00′N, 07°32′E	2	Dry/ *Sphagnum cuspidatum*/*Eriophorum angustifolium*	4.2^b^	0.0	−100	Beyer and Höper (2015)
			Wet/*S. cuspidatum*/*E. angustifolium*	4.2	1.7	−60	
			Deep peat, wet/*S. cuspidatum*/*E. angustifolium*	4.2	0.7	−83	
			Peat extraction/peat mosses cultivation/*Sphagnum papillosum*/*E. angustifolium*	4.2	0.6	−86	
Blanket bog (UK)	52°58′N, 03°49′W	2.3	Rewetted/*Eriophorum vaginatum*	4.5	9.0	100	Cooper et al. (2014)
Fen (NL)	52°11′N, 5°43′E	2	Grasses, reeds and forbs	ND	31.8	n/a	Hendriks, van Huissteden, Dolman, and van der Molen (2007)
Fen (forest; FIN)	MS	1	Restored/forestry	ND	0.9^c^	n/a	Juottonen et al. (2012)
Fen (FIN)	61°48′N, 24°17′E	3	Rewetted/cotton grass	0.1	1.6	>100	Komulainen et al. (1998)
Bog (FIN)	61°51′N, 24°14′E	3	Rewetted/cotton grass	0.6	3.5	>100	
Blanket bog (UK)		0.1	*E. vaginatum*	ND	6.9^c^	n/a	McNamara, Plant, Oakley, and Ostle (2008)
			*S. angustifolium*		2.7^c^	n/a	
			Mixed grasses		0.0^c^	n/a	
			*C. vulgaris*		0.0^c^	n/a	
Treed bog (CA)	47°96′N, 69°42′W	1	Restored field/sedge	6.6	0.4	−95	Strack and Zuback (2013)
			Restored ditch/sedge		15.5	>100	
			Restored site/sedge		1.4	−79	
Bog (CZ)	48°58′N, 13°27′E	2	Rewetted *Trichophorum* spp. lawn	9.5	5.9^c^	−88	Urbanova, Picek et al. (2013)
Bog (CZ)		2	Rewetted high shrub	9.5	1.2^c^	−38	
Bog (DE)	53°44′N, 09°50′E		Rewetted heath	ND	47.8^c^	n/a	Vanselow‐Algan et al. (2015)
Bog (DE)	53°44′N, 09°50′E	1	Rewetted *Sphagnum spp*.		74.7^c^	n/a	
			Rewetted purple moor grass		111.4^c^	n/a	
			Rewetted industrial extraction		0.2^c^	n/a	
Bog (CA)	47°58′N, 69°25′W	4	Restored/peat	0.0^c^	0.0^c^	0	Waddington and Day (2007)
			Restored/moss	5.5^c^	0.0^c^	>−100	
			Restored/shrub	−0.2^c^	0.1^c^	67	
			Restored/herbaceous	−0.1^c^	2.2^c^	>100	
			Restored/ditch	0.1^c^	24.6^c^	>100	
			Cutover/peat	0.0^c^	−0.1^c^	−67	
			Cutover/moss	1.5^c^	0.1^c^	>−100	
			Cutover/shrub	0.1	0.1^c^	0	
			Cutover/herbaceous	−0.2^c^	−0.1^c^	100	
			Cutover/ditch	−0.1^c^	17.9^c^	>100	
Blanket bog (IRE)	54°07′N, 09°35′W	3	Rewetted/*Juncus* spp., *Sphagnum* spp.	0.1^c^	8.2	>100	Wilson et al. (2013)
			Rewetted/*Sphagnum spp*.		9.9	>100	
			Rewetted/*Eriophorum spp*.		5.3	>100	
Treeless bog (DE)	62°12′N, 23°18′E	1	Restored/*Sphagnum riparium*	ND	14.1	n/a	Yli‐Petäys, Laine, Vasander, and Tuittila (2007)

D, duration (years); n/a, not applicable; ND, no data; CA, Canada; CZ, Czech Republic; FIN, Finland; DE, Germany; IRE, Ireland; NL, the Netherlands; UK, United Kingdom; and USA, United States of America.

a Average values were measured/ calculated and converted to g C m^−2^ year^−1^ using original data. A negative CH_4_ value indicates uptake, and a positive CH_4_ value indicates emission.

b Value from Beetz et al. (2013).

c Annual values were estimated from the original seasonal measured values. Methane gas flux during winter was considered as 15% from the annual flux (Maljanen et al., 2010; Saarnio et al., 2007).

As discussed earlier, vascular plants can play an important role in transporting CH_4_ from soils to the atmosphere through aerenchyma (Couwenberg & Fritz, 2012; Henneberg, Elsgaard, Sorrell, Brix, & Petersen, 2015). The establishment of vascular vegetation following extraction is generally more extensive on cutover fens than on cutover bogs (Graf, Rochefort, & Poulin, 2008). Although a combined transportation of O_2_ with CH_4_ by aerenchyma tissues could reduce CH_4_ emissions, previous studies reported higher emissions from vascular plants, especially sedges (Waddington, Roulet, & Swanson, 1996). Roulet, Ash, and Quinton (1993) and Roulet and Moore (1995) reported approximately 23–57 times greater CH_4_ emissions from restored herbaceous vegetation than from a herbaceous vegetation cutover site. Moreover, common cotton grass (*Eriophorum vaginatum*) generates large CH_4_ fluxes (Greenup, Bradford, McNamara, Ineson, & Lee, 2000), whereas peat mosses (*Sphagnum* spp) can act as a CH_4_ sink (Raghoebarsing et al., 2005) following restoration.

Changes with time in CH_4_ patterns after rewetting may be related to previous land use. However, although restoration increases CH_4_ fluxes, it could generally reduce net GHG emissions by reducing CO_2_ flux (Baird, Belyea, & Morris, 2009; Beetz et al., 2013; Samaritani et al., 2011; Strack & Zuback, 2013). Published data on CH_4_ emissions from long‐term (>10 years) rewetted northern peatlands are limited and therefore, changes in gas emissions over time remain uncertain.

The increase in CH_4_ emissions due to restoration must be considered when land use strategies to reduce emissions are developed. Likewise, assessing the suitability of peatland restoration processes requires a better understanding of C processes and dynamics changed by the restoration. Previous studies have resulted in the development of a guide called the “North American Peatland Restoration Guide” (Quinty & Rochefort, 2003). Application of this strategy can result in the return of a plant community that is dominated by species characteristic of peatlands (Quinty & Rochefort, 2003). The new emerging plant community, and altered hydrology resulting from restoration, should help to return GHG dynamics to those more similar to natural peatlands. In addition to producing high CH_4_ emissions, rewetting also increases the dissolved organic carbon (DOC) and thereby the amount of DOC lost to rivers (Dawson & Smith, 2007; Strack, 2008). However, it is still unknown how much is eventually lost to the atmosphere in the form of CO_2_. Thus, to have a clear picture of the advantages/disadvantages of restoration to preserve C stocks of northern peatlands, long‐term investigations on the overall greenhouse gas balance are of great importance.

Future sustainable and climate‐friendly management strategies are needed. These management practices should focus on preventing peatland degradation. Targets of climate protection on managed peatlands could be met by converting arable land to grassland, decreasing land‐use intensity, and re‐establishing the original ground WT (Byrne, Chojnicki, Christensen, Drösler, & Freibauer, 2004; Freibauer, Rounsevell, Smith, & Verhagen, 2004). Petrescu et al. (2015) reported that intensity of land management (e.g., conversion of natural peatlands to agricultural land) can strongly influence net climate footprint of wetlands and could eventually result in positive radiative forcing. They suggested that estimates of future releases of GHG inventories based on IPCC guidelines for wetlands should consider the relationship between CH_4_ and CO_2_ fluxes, the intensity of management, and the land use/land cover change on both the net GHG balance and thereby radiative forcing.

## 5 Conclusions

In this review, we investigated the factors that control CH_4_ emissions and impacts of management in northern peatlands (latitude 40° to 70°N). The study covered a total of 87 studies taken at 186 sites covering different countries, peatland types, and management systems. We found CH_4_ emissions from natural northern peatlands to be highly variable with a 95% CI of 7.6–15.7 g C m^−2 ^year^−1^ for the mean and 3.3–6.3 g C m^−2 ^year^−1^ for the median and an overall annual average (mean ± *SD*) of 12 ± 21 g C m^−2 ^year^−1^. Compared to bogs, fens emit the highest levels of CH_4_ to the atmosphere. The factors controlling the emissions are water table (WT) depth, plant community composition, and soil pH with an interaction with mean annual air temperature, indicating that maximum emissions occurs when MAAT ~ 2°C. Drainage significantly (*p* < .05) reduces the emissions, on average, by 84%, while rewetting of drained peatlands increases the emissions, on average, by 46%. Complex interactions between temperature and the other environmental variables determine CH_4_ emissions from northern peatlands.

## Funding Information

Greenhouse gases Europe project (Grant/Award Number: “244122”).

## Conflict of Interest

None declared.
